# Greenland records of aerosol source and atmospheric lifetime changes from the Eemian to the Holocene

**DOI:** 10.1038/s41467-018-03924-3

**Published:** 2018-04-16

**Authors:** S. Schüpbach, H. Fischer, M. Bigler, T. Erhardt, G. Gfeller, D. Leuenberger, O. Mini, R. Mulvaney, N. J. Abram, L. Fleet, M. M. Frey, E. Thomas, A. Svensson, D. Dahl-Jensen, E. Kettner, H. Kjaer, I. Seierstad, J. P. Steffensen, S. O. Rasmussen, P. Vallelonga, M. Winstrup, A. Wegner, B. Twarloh, K. Wolff, K. Schmidt, K. Goto-Azuma, T. Kuramoto, M. Hirabayashi, J. Uetake, J. Zheng, J. Bourgeois, D. Fisher, D. Zhiheng, C. Xiao, M. Legrand, A. Spolaor, J. Gabrieli, C. Barbante, J.-H. Kang, S. D. Hur, S. B. Hong, H. J. Hwang, S. Hong, M. Hansson, Y. Iizuka, I. Oyabu, R. Muscheler, F. Adolphi, O. Maselli, J. McConnell, E. W. Wolff

**Affiliations:** 1 Climate and Environmental Physics, Physics Institute & Oeschger Centre for Climate Change Research, University of Bern, Sidlerstrasse 5, 3012 Bern, Switzerland; 2 British Antarctic Survey, National Environment Research Council, High Cross Madingley Road, Cambridge, CB3 0ET UK; 3 Centre for Ice and Climate, Niels Bohr Institute, University of Copenhagen, Juliane Maries Vej 30, 2100 Copenhagen K, Denmark; 4 Alfred-Wegener-Institut, Helmholtz-Zentrum für Polar-und Meeresforschung, Am Alten Hafen 26, 27568 Bremerhaven, Germany; 5 National Institute of Polar Research, 10-3 Midori-cho, Tachikawa, Tokyo, 190-8518 Japan; 6 Natural Resources Canada, Geological Survey of Canada, 601 Booth Street, Ottawa, K1A 0E8 Canada; 7 Department of Earth Sciences, Environment and Geomatics, University of Ottawa, Ottawa, ON Canada; 8 State Key Laboratory of Cryospheric Sciences, Cold and Arid Regions Environmental and Engineering Research Institute, Chinese Academy of Sciences, Lanzhou, 730000 China; 9 Institut des Géosciences de l’Environnement, Université Grenoble Alpes, CS 40 700, 38058 Grenoble Cedex 9, France; 10 Institute for the Dynamics of Environmental Processes-CNR, University of Venice, via Torino, 155, 30172 Venice-Mestre, Italy; 11 Korea Polar Research Institute, 26 Songdomirae-ro, Yeonsu-gu, Incheon, 21990 Republic of Korea; 12 Department of Ocean Sciences, Inha University, 100 Inha-ro, Nam-gu, Incheon, 22212 Republic of Korea; 13 Department of Physical Geography, Stockholm University, S-106 91 Stockholm, Sweden; 14 Department of Geology, Lund University, Solvegatan 12, SE-22362 Lund, Sweden; 15 Desert Research Institute, Nevada System of Higher Education, Reno, NV 89512 USA; 16 Department of Earth Sciences, University of Cambridge, Downing Street, Cambridge, CB2 3EQ UK; 17 Present Address: Research School of Earth Sciences, The Australian National University, Canberra, ACT 2602 Australia; 18 Present Address: Fukushima Prefectural Centre for Environmental Creation, 10-2 Fukasaku, Miharu Town, Fukushima, 963−7700 Japan; 19 Present Address: Department of Atmospheric Science, Colorado State University, 200 West Lake Street, 1371 Campus Delivery, Fort Collins, CO 80523−1371 USA

## Abstract

The Northern Hemisphere experienced dramatic changes during the last glacial, featuring vast ice sheets and abrupt climate events, while high northern latitudes during the last interglacial (Eemian) were warmer than today. Here we use high-resolution aerosol records from the Greenland NEEM ice core to reconstruct the environmental alterations in aerosol source regions accompanying these changes. Separating source and transport effects, we find strongly reduced terrestrial biogenic emissions during glacial times reflecting net loss of vegetated area in North America. Rapid climate changes during the glacial have little effect on terrestrial biogenic aerosol emissions. A strong increase in terrestrial dust emissions during the coldest intervals indicates higher aridity and dust storm activity in East Asian deserts. Glacial sea salt aerosol emissions in the North Atlantic region increase only moderately (50%), likely due to sea ice expansion. Lower aerosol concentrations in Eemian ice compared to the Holocene are mainly due to shortened atmospheric residence time, while emissions changed little.

## Abstract (web summary)

Past climate changes in Greenland ice were accompanied by large aerosol concentration changes. Here, the authors show that by correcting for transport effects, reliable source changes for biogenic aerosol from North America, sea salt aerosol from the North Atlantic, and dust from East Asian deserts can be derived.

## Introduction

Greenland ice cores provide unique insight into climate changes over the last glacial cycle, revealing very cold conditions during the Last Glacial Maximum (LGM), relatively stable warm conditions during the Holocene and pronounced rapid warming events during glacial times (Dansgaard Oeschger (D-O) events)^1–3^. Only recently the North Greenland Eemian Ice Drilling (NEEM) ice core^4^ allowed the reconstruction of the first Greenland climate record for the last interglacial period, showing pronounced warming on the Greenland Ice Sheet during the Eemian relative to the Holocene, accompanied by only moderate changes in ice sheet thickness.

Greenland ice core aerosol records also reveal substantial variations in concentrations in the ice accompanying both slow and rapid temperature changes during the glacial^5–10^. Chemical concentrations in the ice are affected by changes in aerosol emissions at the source, changes in atmospheric transport velocity and pathway, as well as changes in aerosol deposition en route and over the ice sheet. Due to this multifactorial control of aerosol concentrations in the ice, it has remained challenging to draw quantitative conclusions from these records about atmospheric concentration and source strength changes. This issue led to diverging interpretations of ice core aerosol records^8,9,11,12^. Some studies^8,13^ concluded that covariant changes in mineral dust and sea salt aerosol in Greenland point to large-scale atmospheric circulation changes being the main reason for their long-term variations. In contrast, other studies based on mineral dust aerosol size distributions and first order deposition estimates concluded that changes in mineral dust aerosol transport pathways were only of secondary importance^12,14^. Only recently, an ammonium (NH_4_^+^) record in annual resolution from the North Greenland Ice Core Project (NGRIP) ice core was used in combination with a simple transport and deposition model to systematically separate emission changes in the North American NH_4_^+^ source region from transport changes^15^.

Here, we investigate high to mid northern latitude environmental and atmospheric changes during the last glacial and the Eemian warm period, using the extended suite of aerosol species analysed in centimetre resolution on the NEEM ice core. We assess the environmental changes implied by the ice core aerosol concentration records using the transport and deposition model approach by ref. ^15^. In particular, we intend to answer the question how much of the strong variations in aerosol concentrations in Greenland ice over glacial/interglacial and stadial/interstadial^16^ transitions are due to changes in atmospheric circulation and precipitation and how much they are influenced by altered emissions. Further, we assess the NEEM aerosol concentration records during the Eemian with respect to potential high-latitude environmental changes connected to the amplified warming signal in high latitudes during that time which is similar to what would be expected in this region for a globally about 1 °C warmer world in the future^17^.

## Results

### Major aerosol species in Greenland ice

Previous studies^15,18,19^ and our extensive back-trajectory study (see Supplementary Fig. 1) indicate that the vast majority of ammonium (NH_4_^+^) (and of soil derived nitrate (NO_3_^−^)) at NEEM originates from North American sources. The NH_4_^+^ found in preindustrial Greenland ice is primarily derived from nitrogen turnover in soils and vegetation as illustrated by the clear summer maximum in NH_4_^+^ deposition at NEEM and other Greenland drill sites^15,20^. Moreover, extraordinarily high NH_4_^+^ peaks during summer in some years are derived from boreal wildfires mainly in North America^15,18,20^. A marine biogenic source is only of secondary importance in Greenland^21^. In the case of NO_3_^−^, additional important sources of nitrate precursors are NO_*x*_ production from lightning at high and mid northern latitudes as well as downward transport of NO from stratospheric oxidation of N_2_O^18,22^.

In Antarctica, sea ice is suggested to be the major source of sea salt aerosol^23–25^, however the relative contributions of the open ocean and sea ice are more difficult to assign for Greenland. A back-trajectory analysis performed for this study (see Supplementary Fig. 2) shows that the vast majority of marine air masses that potentially carry sea salt aerosol (represented by Na^+^) to Greenland originate from the open ocean of the North Atlantic^12^. However, those trajectories are also connected to higher precipitation, hence greater loss en route, than air masses originating from more local marine source regions such as Baffin Bay. Simulations with a 3D-transport-chemistry model^26,27^ suggest that sea ice related processes (especially in the Baffin Bay) contribute up to 50% to the recent winter maximum in sea salt aerosol concentrations at NEEM. In summary, we conclude that a large part of the sea salt aerosol in Greenland today comes from the open North Atlantic, however sea ice, especially in Baffin Bay, represents a significant additional winter source, which is expected to become relatively more important in glacial times, when sea ice largely expanded.

According to mineralogical and Sr/Nd isotopic studies, mineral dust aerosol at Greenland deep ice core sites originates mainly from East Asian desert regions both for interglacial as well as glacial conditions^28,29^. Given that the major source region does not change substantially over time, it is reasonable to assume that also the elemental composition of the mineral dust aerosol mobilized at the source did not change substantially. Here, we use high-resolution Ca^2+^ data to quantify past changes in mineral dust aerosol. From the East Asian source regions mineral dust derived Ca^2+^ is efficiently uplifted by high wind speeds during dust storms into the upper troposphere (ref. ^30^ and references therein) in boreal spring, allowing for efficient long-range transport to the Greenland ice sheet. The remote source location of mineral dust derived Ca^2+^ and its transport at high altitudes above cloud-level imply longer transport times and reduced wet deposition en route^31–33^ compared to the other major aerosol species found in Greenland ice (see Methods). Mineral-dust derived Ca^2+^ (which is partly soluble) may have a somewhat different atmospheric residence time compared to particulate dust. This could lead to different relative changes in Greenland ice core concentrations, however, long-term changes in the Ca^2+^/dust ratio in Greenland ice are typically smaller than a factor of 2 (ref. ^34)^, i.e., much smaller than the observed Ca^2+^ concentration changes.

Finally, preindustrial background concentrations of sulfate (SO_4_^2−^) aerosol in Greenland are mainly derived from Northern Hemisphere marine biogenic (dimethyl sulfide) and non-eruptive volcanic emissions^35,36^. In addition, volcanic sulfur emissions into the stratosphere strongly influence sulfate concentrations in the years directly following major eruptions^37^. Only a small terrestrial contribution to current Greenland SO_4_^2−^ levels exists, but may have varied somewhat on glacial/interglacial time scales^36,38^.

**Fig. 1:**
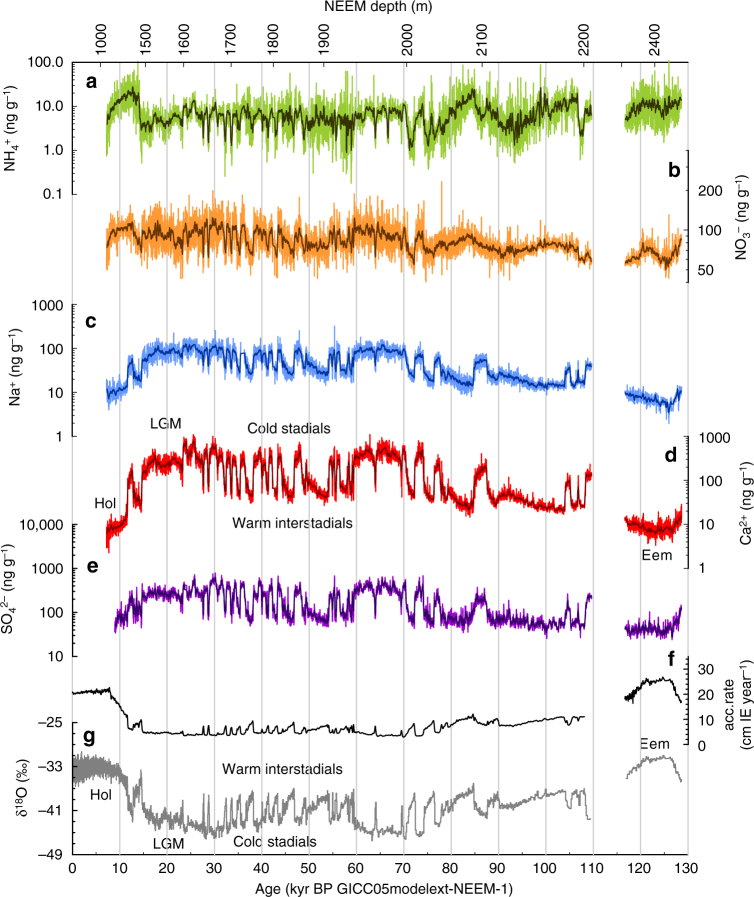
Measured ion concentrations in the ice. Variations of the aerosol concentration records in the ice in 10-year resolution (light coloured lines) for **a** NH_4_^+^, **b** NO_3_^−^, **c** Na^+^, and **d** Ca^2+^ (note the smaller logarithmic range of the NO_3_^−^ axis). In the case of SO_4_^2−^ (**e**) the light thin line represents a five point running mean of the 10 year data, representative of the maximum 40 year resolution that can be achieved with the SO_4_^2−^ data over the entire length of the record. The dark bold lines represent 21 point running means of the 10 year data. The record of the glacial inception is missing due to folding in the ice^4^. In addition, the accumulation rate (black line) is plotted in **f** for 0–108 kyr BP according to ref. ^62^. For the Eemian interval the accumulation is based on an exponential fit of the north west Greenland accumulation and δ^18^O relationship^54^. In **g** NEEM δ^18^O is shown as a grey line in 55 cm resolution for the time period 0–110 kyr BP and for the Eemian period^4^. All data are shown on the GICC05modelext-NEEM-1 age scale^62^ in years BP

Here we present ion concentration records from the NEEM ice core for NH_4_^+^, NO_3_^−^, SO_4_^2−^, sea salt aerosol (Na^+^) and mineral dust aerosol (Ca^2+^) based on continuous melt water analyses (see Methods section). The records essentially cover the time interval from the Eemian to the early Holocene period in 10-year resolution. In line with previous studies, all aerosol concentrations in the ice exhibit millennial-scale variability in parallel to local temperature changes as indicated by δ^18^O of the ice (Fig. 1), but with different amplitudes for the different aerosol species. Calcium shows the highest glacial concentration increase by a factor of 40–80 compared to the early Holocene. In comparison, the glacial increase of Na^+^ and SO_4_^2−^ in the ice is about a factor of 10. Moreover, Ca^2+^, Na^+^, and SO_4_^2−^ all show pronounced decreases during warm interstadial events. In contrast, NO_3_^−^ and NH_4_^+^ concentrations in the ice remain the same or are even reduced during glacial times compared to the Holocene and show little stadial/interstadial variability.

### Past changes in aerosol emissions and transport

Applying a simple mean aerosol transport model^9,11,12,15^, we evaluate the contributions of emission changes and transport and deposition effects to the measured aerosol concentration in Greenland ice both for the significant long-term glacial/interglacial concentration changes and for rapid D-O events. The loss of aerosol en route is dependent on the transport time from its source to NEEM and the atmospheric residence time which is controlled by dry and wet deposition en route, hence precipitation. The main factors taken into account in the model approach (see Methods section) are temporally changing precipitation at NEEM, which locally affects the deposition of the ion species, and temporally changing precipitation en route, hence atmospheric residence time, during transport from the source region to the ice sheet. As the precipitation rate en route for the past is not known from paleodata, we assume that it linearly scales at first order with changes in accumulation at NEEM (see Methods section), assuming that large scale circulation changes from the source to Greenland control the precipitation at NEEM and en route. Correcting for these temporal changes in aerosol deposition, we can derive the net changes at the source potentially related to factors as changes in aridity, wind speed or biogenic activity.

Changes in transport time from the source to NEEM are controlled by potential changes in glacial wind speeds relative to modern times on the one hand and larger glacial transport distance due to expansion of continental ice in North America and multiyear sea ice cover in the North Atlantic region on the other. Observational evidence for past wind speed is rare. Atmospheric models suggest a relatively small surface wind speed change on a global average^39^, while regional changes in wind speed may have occurred. Somewhat higher glacial wind speeds are modelled in the mid latitude North Atlantic region as well as a switch of the jet stream from a glacial strong zonal to a more meridionally tilted interglacial configuration^40^. However upstream of Greenland, wind speed changed only little^40^. Overall the magnitude of regional wind speed changes is on the order of tens of percent, while the expansion of the continental ice sheets and sea ice is very large, effectively suppressing a huge part of recent continental and open ocean source areas in the glacial. Accordingly, the about two times larger distance from the aerosol source to Greenland is likely to dominate the transport time variations. In our best-guess model runs we assumed a factor of ~2 longer glacial transport time for continental NH_4_^+^ and marine Na^+^, reflecting the expansion of the Laurentide ice sheet and North Atlantic permanent sea ice cover, respectively. In addition, we performed sensitivity runs to assess the effect of varying transport time.

**Fig. 2:**
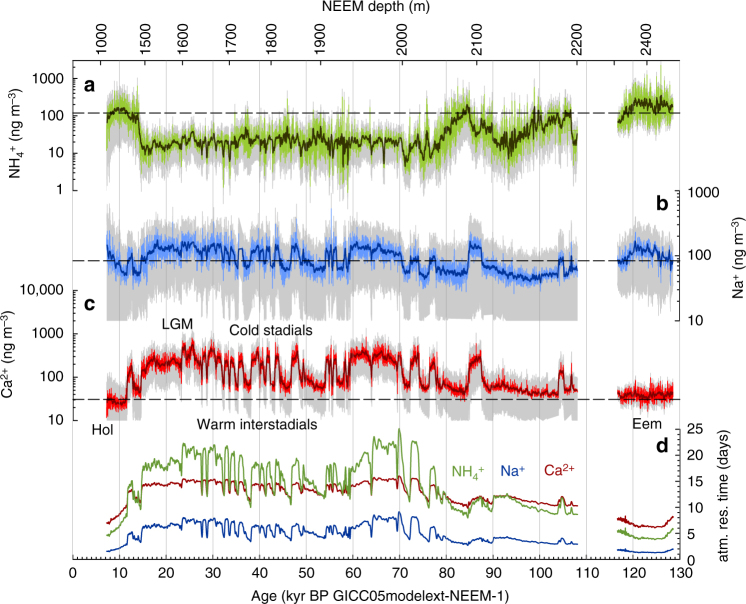
Reconstructed source concentrations. Variations of the source concentration records in 10 year resolution (light coloured lines) for **a** NH_4_^+^, **b** Na^+^, and **c** Ca^2+^ (note the smaller logarithmic range of the Na^+^ axis). The dark bold lines represent 21 point running means of the 10 year data. The dashed straight line indicates the early Holocene average. The grey area represents the uncertainty band (1 sigma) as calculated from Gaussian error propagation of the deposition parameters. Note that in the error propagation for the Eemian section only the uncertainty in the past precipitation rate is included, while we assumed that the other deposition parameters in the model were the same for the Eemian and the Holocene and, thus, do not introduce an additional error when comparing Eemian and Holocene values. In panel **d** the calculated atmospheric residence time for each of the aerosol species is plotted

For mineral dust aerosol, provenance studies^29,41^ showed that the source region, hence the transport distance to Greenland, remained essentially the same in the glacial and interglacial. Atmospheric models find that the jet stream may have been seasonally somewhat stronger than present^40^ and may have been split around the Laurentide Ice Sheet during the LGM^42^, however, wind speed changes were much smaller than a factor of 2. Another possibility for a change in dust transport time could be a contribution of glaciogenic dust on the North American continent in glacial times. Dust model runs show an improved agreement with Greenland dust deposition when tuning glaciogenic dust source strength in the US great plains for glacial conditions^43^, however, these sources were previously rejected based on the isotopic signature of dust in Greenland ice cores^28,29^. In summary, in our best-guess scenario we assumed the location of the main dust source region and the transport time from the East Asian desert regions to NEEM to stay constant in time, but performed sensitivity studies for a very wide range of transport time scenarios (see Supplementary Fig. 5). Translating our concentrations in the ice into atmospheric aerosol concentration changes at the source (Fig. 2) provides evidence of climate-induced variations in the environmental conditions in the source regions of the different aerosol species. The difference in amplitude of the relative changes in ice and source concentrations (Supplementary Fig. 3) reflects the changing deposition effects en route and over the ice sheet.

The terrestrial biogenic aerosol species NH_4_^+^ shows nearly ten times higher values for the atmospheric concentration at the source during full interglacial periods (the Eemian and the Holocene) compared to the glacial (Fig. 2) but also similarly high values during later Marine Isotope Stage (MIS) 5 warm periods (around 85 kyr before present (BP, where present is defined as 1950) and 98–106 kyr BP in the NEEM record). During that time of glacial inception sea level was still high^44^, hence continental ice volume was low. In contrast, source concentrations point to drastically reduced emissions during the glacial. Mean glacial NH_4_^+^ source strength during the time interval 15–70 kyr BP, corresponding roughly to MIS 2–4, is decreased to about 10 ± 10% of its early Holocene value (Fig. 2 and Supplementary Fig. 3). This glacial reduction can be attributed to the diminished biological activity on the North American continent, partly due to reduced temperatures but mainly because of the huge expansion of the Laurentide ice sheet and the consequent shrinkage of vegetation covered area which effectively suppresses terrestrial biogenic aerosol production^15,20,45^. In addition, any marine biological NH_4_^+^ aerosol production may have been even further suppressed compared to today by the glacial increase in multi-year sea ice^46,47^.

NO_3_^−^ shows a similar behaviour (Supplementary Fig. 4) but interpretation of NO_3_^−^ in terms of source changes is ambiguous as gradual formation of NO_3_^−^ aerosol en route from gaseous precursors is not included in our simple transport model. However, overall reduced glacial NO_3_^−^ formation is in line with lower terrestrial biogenic emissions and reduced lightning activity in the middle and high latitudes as expected from the reduced convective activity during cold climate periods.

During times of expanded Laurentide ice sheet cover, the NH_4_^+^ baseline signal in source concentrations shows no clear millennial-scale D-O variability (Fig. 2). However, a significantly higher frequency of intermittent NH_4_^+^ peaks from wildfires was recorded in annual resolution data from the Greenland NorthGRIP ice core during warm interstadial and interglacial conditions, in line with an increased ignition risk during those times^15^. Millennial changes in glacial NO_3_^−^ source concentrations (Supplementary Fig. 4) are very subdued without any visible influence of rapid D-O events, in line with a small contribution of wildfires to NO_3_^−^ concentrations in Greenland^19^.

Due to the extended sea ice cover and the topographic effects of the Laurentide ice sheet during cold glacial conditions^48,49^, we expect a southward displacement of sea salt aerosol formation from the open ocean in glacial times and a return northwards toward Greenland during major warmings^50^. The same is true for sea salt aerosol derived from sea ice as an expansion of multi-year sea ice around Greenland should suppress efficient sea salt aerosol formation. Accordingly, we used an approximately two times higher glacial transport time compared to the Holocene to mimic this in our best-guess run. Even with this larger glacial transport time, this leads to a reconstructed glacial/interglacial emission decrease for sea-salt aerosol (Na^+^) that is markedly smaller than the change directly measured in the ice. While Na^+^ concentrations in the ice change by a factor of up to 10 between glacial and interglacial, the emissions of sea-salt aerosol during colder periods were increased by only a factor of about 1.5 (Fig. 2 and Supplementary Fig. 3) with a likely range between 1 and 2. Much stronger increases in glacial source concentrations are not supported by our model. The strong difference in glacial/interglacial changes in ice concentrations and reconstructed source concentration clearly shows that interpretation and use of uncorrected ice concentrations can lead to misleading conclusions and that covariance in ice concentrations between different aerosol species may largely reflect parallel changes in aerosol deposition en route. Note that assuming the same transport time in the glacial as today (the latter being determined from back-trajectory studies for recent conditions) leads to slightly decreased glacial emissions compared to the early Holocene. In light of this, reduced glacial emissions relative to the early Holocene cannot entirely be ruled out using our model approach but are less likely. We attribute the 50% increase in source concentrations in our best-guess run to a net enlargement of the sea ice source despite growing multi-year sea ice. According to our model the emissions during interstadials were lower than during stadials and similar or slightly reduced compared to early Holocene values. However within our model uncertainties, constant stadial/interstadial Na^+^ source concentrations cannot be ruled out. In summary, transport and deposition effects appear to control a large part of the observed variability of Na^+^ concentrations in the ice leading to a relatively small increase in glacial sea salt aerosol emissions. A more detailed assessment of the relative importance of source and transport effects for sea salt aerosol requires dedicated atmospheric circulation model and process studies of aerosol formation over the open ocean and over sea ice in the North Atlantic.

We also applied our transport model to the Ca^2+^ concentrations in the NEEM ice core, taking the different transport properties of Ca^2+^ compared to the other aerosol species (longer transport time, increased transport height, see Methods section) into account. Due to the much lower precipitation rate en route assumed for Ca^2+^ along its upper troposphere transport pathway from East Asian desert regions to Greenland, much stronger temporal changes in source strength are reconstructed by our simple model compared to sea salt aerosol. The Ca^2+^ source concentration, reconstructed assuming that East Asian desert regions were always the major source of mineral dust aerosol in Greenland, is enhanced considerably by up to a factor of about 8 in the Last Glacial Maximum (LGM) compared to the early Holocene in line within the uncertainty with mineral dust aerosol flux changes recorded in a northern Pacific sediment core downwind of Asian dust sources^51^. Stadial emissions are increased by up to a factor of 10 relative to the early Holocene and by about a factor of 4 relative to interstadials (Fig. 2, Supplementary Fig. 3). The amplification factors given above are dependent on the transport time and precipitation en route used in the model, however, sensitivity studies (Supplementary Fig. 5) show, that even an unrealistically short transport time during the glacial of only 50% of the Holocene value does not change our overall conclusion on significant changes in dust emissions between warm and cold periods. Also assuming three times higher wet scavenging en route for dust transport above cloud level, hence an atmospheric residence time roughly 50% of our best-guess value, does not change the conclusion that substantial source changes in mineral dust mobilisation must have occurred. Assuming, however, a much higher precipitation rate (similar to the one for sea salt aerosol (Na^+^), i.e., representative of dust aerosol transport below cloud level), would imply much smaller source changes. In view of current observations of mineral dust mobilization in East Asian dust areas and its long-range transport to Greenland at high altitudes, such high precipitation rates are, however, very unlikely.

The results imply that transport/deposition effects contributed to the 40–80 fold Ca^2+^ concentration increase in the ice by at least a factor of ~5 over the last glacial cycle. Thus according to our idealized transport model, Ca^2+^ concentration changes in the NEEM ice core are primarily attributed to changes in the source strength in the East Asian desert regions with only a secondary albeit sizable influence of transport related changes (see Methods section for additional sensitivity experiments). In line with previous work^7,12,52^, we propose that glacial environmental changes involved strongly enhanced dust storm activity with (at least seasonally) higher wind speeds and enhanced gustiness over the East Asian desert regions, increased aridity related to changes in the Asian monsoon system^34^, and/or a shift in position or strengthening of the jet stream over East Asia during cold climate conditions.

Using the same simple transport model approach for SO_4_^2−^, the reconstructed changes in sulfate aerosol show little change in peak glacial source concentrations compared to the early Holocene—as was the case for sea salt aerosol—but a more pronounced stadial/interstadial variability—as seen in mineral dust aerosol (Supplementary Fig. 4). This observation may be linked to the combination of reduced marine biogenic SO_4_^2−^ emissions during glacial times and the variable uptake and neutralization of acidic SO_4_^2−^ aerosol on mineral dust particles in the atmosphere. This neutralization reaction is dependent on the abundance of alkaline mineral dust aerosol in the atmosphere, which strongly changes between stadials and interstadials. In contrast, the Ca^2+^/SO_4_^2−^ mass ratio of approximately one for those stadial/interstadial changes excludes a direct injection of SO_4_^2−^ containing mineral dust particles to be responsible for the strong SO_4_^2−^ changes. Note that such an uptake of SO_4_^2−^ during transport of mineral dust aerosol is not included in our transport model and neither is the production of SO_4_^2−^ by oxidation of gaseous precursors en route. This leads to potentially varying extensions of the effective atmospheric residence time on stadial/interstadial timescales and interpretation of the reconstructed SO_4_^2−^ source concentrations may be biased by this effect. In summary, the formally reconstructed SO_4_^2−^ source concentrations cannot be interpreted as emission changes of a well defined source region but reflect the interplay of different sources and aerosol chemical reactions in the atmosphere.

### Environmental conditions during the warm Eemian period

**Fig. 3:**
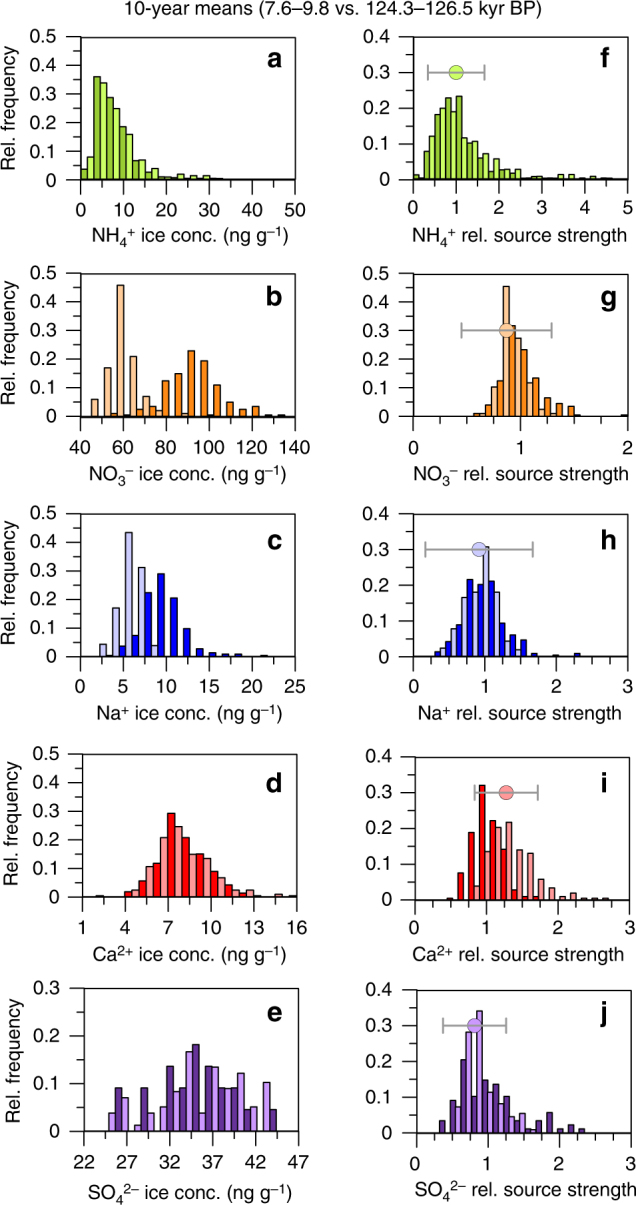
Comparison of Eemian and Holocene concentrations. Histograms of 10-year means in the concentrations in the ice for **a** NH_4_^+^, **b** NO_3_^−^, **c** Na^+^, **d** Ca^2+^, and **e** SO_4_^2−^ for the early Holocene reference period (dark colors) and the Eemian reference period (light colors). **f**–**j** shows histograms of the reconstructed source concentration for these aerosol species in 10 year resolution relative to the early Holocene mean for the early Holocene reference period (dark colors) and the Eemian reference period (light colors). The dots indicate the median of the reconstructed relative source concentrations for the Eemian period. The error bars indicate the median of the uncertainty of the calculated relative source concentrations, where only the uncertainty in the past precipitation rate is included in the error propagation assuming that the other deposition parameters were the same for the Eemian and the Holocene. Note that in contrast to Figs. 1 and 2 the accumulation rate used to quantify the wet deposition during the early Holocene is also based on an exponential fit of the north west Greenland accumulation and δ^18^O relationship^54^

In addition to the glacial/interglacial and stadial/interstadial variations in environmental conditions recorded in the NEEM aerosol concentration records, we are now for the first time able to compare aerosol concentration records from the Eemian interglacial period in Greenland ice with the early Holocene. The Eemian is characterized by similar or lower concentrations in the ice in all five aerosol species, with particularly pronounced reductions in Na^+^ and NO_3_^−^ (Figs. 1 and 3). Taken at face value this could be interpreted as a significant change in NO_3_^−^emissions but surprisingly little change in the other biogenic aerosol species NH_4_. In contradiction to our expectation for warmer climate conditions, the Na^+^ decrease could be erroneously used to conclude that the Eemian was characterized by reduced storminess or a higher ice sheet (as atmospheric sea salt aerosol concentrations typically show an exponential decline with altitude^53^). However, we attribute the observed concentration changes in the ice mainly to the higher precipitation rate en route (see below and the Methods section for estimates of past precipitation rate) during the Eemian, which led to enhanced wet deposition during transport.

Assuming a mean accumulation rate ~25% higher during the early Eemian compared to the early Holocene value (both accumulation rate estimates based on the current spatial temperature/accumulation relationship in Northwest Greenland^54^) due to higher Eemian temperatures, our transport model predicts also higher precipitation rates en route. Correcting for the accompanying transport deposition effects shows that the median in Eemian NH_4_^+^ and NO_3_^−^ source concentrations (Fig. 3) remained essentially the same as the early Holocene value. From these results we conclude that the North American biogenic source was not significantly stronger during the Eemian compared to the early Holocene, despite warm conditions in high latitudes. Unfortunately, due to the strong thinning of Eemian ice by glacier flow, the temporal resolution of the NH_4_^+^ record is not sufficient to unambiguously detect single wildfire peaks during the Eemian period. Accordingly, an assessment of Eemian vs. Holocene wildfire activity is not possible at this point. For SO_4_^2−^ source concentrations, whose marine biogenic contribution may be different during warmer climate conditions in the Eemian, a decrease to 80% of the Holocene value is suggested, however, this decrease is not significant within the uncertainty of our reconstruction.

Due to the higher scavenging efficiency for Na^+^, the effect of changes in precipitation en route in our model on Na^+^ source concentration is very pronounced (Fig. 3). In contrast to the concentration in the ice, median Na^+^ source concentration during the Eemian tends to be decreased only slightly to 90% of the early Holocene value. However, similar to the biogenic species, the uncertainty of our reconstruction also allows for unaltered or slightly higher Na^+^ source concentrations. In particular, the uncertainty in the reconstruction of Eemian snow accumulation and precipitation en route, does not allow us to make inferences about ice sheet elevation or sea ice changes. Nevertheless, the overall small Eemian/Holocene changes in the aerosol species are in agreement with very similar zonal wind speed and an unaltered location of the jet stream during the early Holocene and the Eemian as modelled in a general circulation model^55^.

For the mineral dust aerosol tracer Ca^2+^, we reconstruct moderately elevated source concentrations in the Eemian compared to the early Holocene (by a factor of 1.3). This may suggest slightly higher dust emissions in the East Asian desert regions, potentially related to increased aridity or uplift.

In summary, the reductions observed in Eemian ice core concentrations largely disappear when the higher wet scavenging in the Eemian is taken into account. The remnant environmental changes reflected in aerosol source changes during the Eemian are moderate, even though the Eemian was significantly warmer in the Arctic, and globally showed significant warming and elevated sea level^56–58^. Despite the different causes of the Eemian warming (different orbital parameters but similar greenhouse gas concentrations compared to preindustrial) and the present anthropogenic climate change (high greenhouse gas concentrations at the essentially same orbital parameters as preindustrial), the Eemian temperature distribution looks similar to the polar amplification signal expected for a global warming of 1.5–2 °C by the end of the 21st century^17,59^. Allowing for caveats in rates of change and transient vs. equilibrium climate response, our records may serve as a first order estimate of environmental changes in the mid to high latitude Northern Hemisphere at 2100. Accordingly, our records suggest that keeping global warming below 1.5–2.0 °C will likely avoid drastic changes in the environmental factors in this region controlling the formation of the aerosol species investigated in this study.

## Methods

### Ice core analyses

From 2008 to 2012 the NEEM deep ice core in north-western Greenland (77.45°N, 51.06°W, mean annual temperature −29 °C, modern accumulation 22 cm ice equivalent (IE) per year) was drilled and recovered to a depth of 2540 m (ref. ^4^). The NEEM core shows clear stratigraphic disturbances in the bottom part due to folds in the ice. However, the stratigraphy was successfully restored^4^, resulting in the first dated Eemian climate record from a Greenland ice core. The folding in the ice results in replicate sections of ice from certain age intervals (107–119 kyr BP, also clearly seen in the aerosol records) and in a gap (110–114 kyr BP) in the records due to missing ice in the vertical profile of the Greenland ice sheet at NEEM (Fig. 1).

Here we present results from the chemical analyses performed on the NEEM ice core below the brittle ice zone using the University of Bern Continuous Flow Analysis (CFA) system^60^ extending back to 128 kyr BP. We obtained continuous CFA records of Na^+^, Ca^2+^, NH_4_^+^, and NO_3_^−^ of the depth section from 1178 to 2432 m, corresponding to ages from 7.2 to 128.6 kyr BP with a gap between 109.6 and 116.6 kyr BP. The records feature an unprecedented resolution of 10 years throughout the entire records. 110 cm long ice rods with a square cross section of 36 × 36 mm were continuously melted with a typical melt speed of 3.5 cm/min on a square melter head. The relative concentration errors are typically smaller than 10% for all species^61^. In the case of Ca^2+^, the use of a new drilling fluid (Coasol/Estisol mixture) at NEEM led to sizable adsorption/desorption effects in the CFA system. This implies that the Ca^2+^ concentration at the beginning of each 110 cm run was systematically too low but approached the true mean concentration within several tens of centimeters. We used an empirical correction to remove this artificial trend in each run by assuming that the mean of the second half of each run is representative for the entire run (see Supplementary Fig. 6 for a typical example of the data before and after correction). This implies that the variations on depth scale of tens of centimeters within one run should not be interpreted in terms of atmospheric changes but that the mean over each run is correct. Accordingly, the multi-decadal to millennial variations interpreted in this study are not affected by this correction, as also shown by the very good correspondence (Supplementary Fig. 6) of the corrected decadal NEEM data with the NGRIP Ca^2+^ record^16^. Nevertheless other users are encouraged to use the NGRIP Ca^2+^ data available in 20 year resolution, which is not affected by this drill fluid effect.

In addition to the measurements incorporated in the CFA analysis system (electrolytic conductivity, Ca^2+^, Na^+^, NH_4_^+^, NO_3_^−^, H_2_O_2_, and insoluble particles) other analytical systems were connected to the melthead and sample distribution system. These comprised: Fast Ion Chromatography (FIC) (capable of discrete NO_3_^−^, SO_4_^2−^, Cl^−^, MSA, and F^−^ analysis); a Single Particle Soot Photometer (SP2) to detect black carbon particles; and various sampling vials to collect the uncontaminated part of the waste lines for additional discrete low-resolution Ion Chromatography (IC), Coulter Counter (CC), inductively coupled plasma mass spectrometry (ICPMS), and tephra measurements. The British Antarctic Survey FIC system used two discrete anion chromatography channels with rapid isocratic elution through columns held at high temperature to reduce eluent viscosity and column pressure. The full suite of anions was eluted in 2.7 min, while interlacing of the two channels allowed a sample to be injected every 1.35 min. Continuous loading of one channel or the other from the melthead liquid flow implies that the depth resolution was 1.35 times the melt speed (i.e., typically a 5 cm depth resolution). We report here the sulfate data from a single channel through the whole section, i.e., approximately an average over 5 cm of ice every 10 cm of ice.

NEEM CFA data is available in 1 mm resolution. However, due to sample dispersion in the system, analytic response time, and the melt speed, the effective resolution is reduced^60^ to 12 mm for Ca^2+^, 19 mm for NH_4_^+^, and 26 mm for Na^+^ and NO_3_^−^. For this study the high-resolution data is down-sampled to 10 year means according to the GICC05modelext-NEEM-1 age scale^62^. This corresponds to typically 50–60 cm of ice during the Holocene, 10 cm during the peak glacial (~14–28 kyr BP), 15 cm during interstadials and 5–10 cm during stadials in the time interval 28–59 kyr BP (corresponding roughly to MIS3). In the time interval 59–70 kyr BP (equivalent to MIS4) the layer thickness is further reduced due to low accumulation and thinning, resulting in 10 year means which are recorded within 3–4 cm of ice, while it is again more than 5 cm from 70 to 96 kyr BP. Due to a kink in the layer thickness of the age scale at 96 kyr BP^62^, 10 years correspond to only 1–2 cm between 96 and 110 kyr BP. In the Eemian warm period (equivalent to MIS5e), 10 years correspond to 2–10 cm of ice^4^. Thus, with the resolution of our CFA, we achieve 10 year resolution throughout the entire NEEM ice core except for the time interval 96–110 kyr BP, where the resolution, however, is still better than 20 years. For the FIC single channel sulfate data, the maximum resolution that can be achieved over the entire length of the core is only about 40 years, however decadal resolution is possible over an extended time period between the Holocene and 60,000 years BP. We provide a record in 40 years resolution in Fig. 1.

Na^+^ is largely derived from sea salt aerosol in Greenland, however, there may be a small contribution of Na^+^ from mineral dust in occasionally occurring thin layers with very high dust content. Here we use only 10 years averages, which are not significantly affected by a mineral dust contribution. Vice versa, Ca^2+^ is dominated by mineral dust sources, but also a small sea salt contribution cannot be excluded. The sea salt contribution to Ca^2+^ has been estimated using the mean Na^+^ concentration in the ice and the Ca^2+^/Na^+^ ratio in sea water to be around 10% in the Holocene but much less in the glacial, where very high mineral dust levels prevail^12^. In view of this small contribution, we refrain from a sea salt correction of our Ca^2+^ data.

### Mean trajectory transport and deposition model

To simulate the deposition process we followed the simple approach by Fischer et al.^15^ and applied this deposition model (as depicted in Supplementary Fig. 7) including dry and wet deposition^9,11,12^. This model translates the aerosol concentration in the ice (*C*_ice_) back to the concentration in the atmosphere (*C*_air_) above the deposition site according to:

1 $$C_{{\mathrm{air}}}\, = \,\frac{A}{{A\cdot \varepsilon _{\mathrm{s}} + v_{{\mathrm{dry}}}}}C_{{\mathrm{ice}}}$$

where *A* is the accumulation rate, *ε*_s_ is the scavenging ratio in snow, and *v*_dry_ is the dry deposition velocity.

The reconstructed atmospheric concentration is then used as starting point to calculate the concentration in the air at the source (*C*_0_) of the respective component. We use a simple model of aerosol atmospheric transport along a mean transport air mass trajectory assuming an exponential decrease of the concentration in the air parcel on its way to the ice sheet^9,12,15^ leading to:

2 $$C_0\, = \,C_{\mathrm{air}} \cdot {\mathrm{e}}^{\frac{t}{\tau }}$$

$${\mathrm{with}} \,\tau \, = \,\frac{h}{{v_{\mathrm{dry}} + P\varepsilon _{\mathrm{r}}}}$$

Here, *t* is the transport time and *τ* the atmospheric residence time of the aerosol with *h* the typical thickness of the air column carrying aerosol along its trajectory to Greenland and *P* the precipitation en route. For the dry deposition velocities *v*_dry_ and scavenging ratios en route *ε*_r_ (representative for rain) and over the ice *ε*_s_ (representative for snow) we used typical literature values listed in Supplementary Table 1. Note that the atmospheric lifetime of the aerosol in this simple model is largely controlled by the mean precipitation en route. In principle, temporal changes in the precipitation frequency could also have a significant influence on aerosol lifetimes, however, as we have no paleo information on the frequency of precipitation events en route, we use only the mean precipitation to effectively parameterize the atmospheric lifetime. Given the good agreement of the calculated modern lifetimes in our model with literature values, this seems justified, but leaves room for improvement in future research.

Based on this model we performed a systematic Gaussian error propagation and performed sensitivity runs to constrain the uncertainty of this reconstruction and their impact on our conclusions. The uncertainty of the deposition effect is dependent on the deposition velocity, hence also on the scavenging ratio, which has a large uncertainty itself. This implies that the uncertainty of the reconstruction is generally large and in the case of Na^+^ of the same order as the observed glacial/interglacial and stadial/interstadial variations. For NH_4_^+^, however, where only glacial/interglacial variations in the source concentrations are observed, the uncertainty is significantly smaller than the observed long-term changes. The same holds true for Ca^2+^, where also stadial/interstadial changes in the source strengths are much larger than the uncertainty.

We assume a constant column thickness *h* for NH_4_^+^, NO_3_^−^, Na^+^, SO_4_^2−^, and Ca^2+^ of 4 km but also used 8 km in a sensitivity run for Ca^2+^ (Supplementary Fig. 5), the latter leading to somewhat higher source concentrations changes relative to the early Holocene. The mean annual precipitation along all back-trajectories starting at NEEM in the time interval 1980–2010 was used as average modern precipitation at ground level *P*_0_ separating trajectories originating in the terrestrial (representative for NH_4_^+^ and NO_3_^−^) and marine (representative for Na^+^, SO_4_^2−^) boundary layer. This led to *P*_0,terr_ = 815 mm water equivalent (WE)/year and *P*_0,mar_ = 1288 mm WE/year for terrestrial and marine trajectories, respectively. For sea salt aerosol this translates to a modern atmospheric residence time of 1.6 days, in line with the wet deposition lifetime in global circulation models^39^. Since precipitation in the upper troposphere above cloud level is strongly suppressed, we reduced the effective precipitation rate for Ca^2+^ to *P*_0,dust_ = (0.1 ± 0.1) ·*P*_0,terr_. This leads to modern atmospheric residence times of 7.3 days, in very good agreement with global circulation model studies^43^. In addition, we performed a sensitivity run where we reduced the current precipitation rate en route for dust only to one third of the below-cloud value (see discussion below and Supplementary Fig. 5).

For past precipitation rate en route *P* we simply assume that it scales linearly in time with the accumulation rate at NEEM, e.g., if accumulation at NEEM is only half of the modern accumulation, we assume the mean precipitation rate en route to be only half of the modern precipitation rate *P*_0_. In contrast to estimates using the Clausius–Clapeyron relationship to derive past precipitation rates, we believe that our simple assumption is more realistic as it inherently takes into account that the past precipitation changes are also strongly affected by dynamic and not only thermodynamic changes^50^.

For the modern transport time, we use the average time of all back-trajectories (using ERA interim reanalysis data) starting at NEEM to reach the boundary layer at the source region for the first time. These mean values are ~5.7 days for Na^+^ and SO_4_^2−^ (main trajectory source region is the North Atlantic and adjacent basins east and west of Greenland between 40° and 75°N, while transport to NEEM from the Artic basin is relatively rare) and 6.4 days for NH_4_^+^ and NO_3_^−^ (main source region is North America between 40° and 70°N). The mean values vary somewhat dependending on assumed boundary level height and altitude of the back-trajectory starting level above the NEEM site. We include this variability in the uncertainty estimate (1 day) of our transport time. In the case of Na^+^, which is both of open ocean and sea ice origin, the transport time to NEEM for the modern sea ice source in winter (mainly located in the Baffin Bay) is shorter than 5.7 days, however, the number of trajectories from the Baffin Bay to NEEM has only a small influence on the mean annual transport time. To study the potential influence of the range in transport times we performed sensitivity runs displayed in Supplementary Fig. 5 and discussed below.

For Ca^2+^ from East Asian dust origin it is not possible to estimate the current transport time using back-trajectories, because very long back-trajectories become increasingly uncertain and the back-trajectories will very rarely enter the boundary layer in the Asian desert source regions. In fact, a maximum 10-day back-trajectory analysis for the NEEM site showed very little chance for air parcels to originate from East Asian desert regions (see Supplementary Fig. 1). In the case of dust, the terrestrial aerosol is uplifted by strong surface winds at the Asian source and then, aided by the surface topography, directly injected into the upper troposphere; processes not well reflected in reanalysis data. After uplift, the dust is transported by fast zonal winds at high altitudes (connected to the jet stream) eastward toward North America and then Greenland. In line with satellite observations^32,33^ and forward transport modeling, such high troposphere mineral dust aerosol plumes originating from the Taklamakan and Gobi deserts and being transported eastward need typically 10–13 days to reach the longitude of Greenland^31^. Accordingly, we assume a mean transport time of 11.5 ± 1 day for Ca^2+^ substantially larger than for the North American source of NH_4_^+^ and NO_3_^−^. In addition, we performed sensitivity runs where we changed the transport time significantly compared to our best guess of 11.5 day.

During the last glacial period, wind strength between mid and high latitudes may have been enhanced through more pronounced meridional pressure and temperature gradients. This would lead to faster (zonal) circulation and enhanced (meridional) eddy mixing, hence to a potential decrease in transport time *t*. However, the source regions in glacial conditions for NH_4_^+^, NO_3_^−^, and sea salt aerosol (Na^+^) and SO_4_^2−^ from the ocean surface were shifted southward^50,63^, i.e., away from Greenland, which leads to a generally longer transport time *t*. It is very difficult to quantify these two contrary effects without dedicated 3D modelling studies, however, in view of the vast expansion of continental ice cover in North America and in sea ice cover around Greenland and over the North Atlantic, the longer transport distance is likely to dominate changes in the transport time and in our best-guess estimate we assume a two-fold increase in transport time under glacial conditions.

In our sensitivity runs, we kept transport time *t* constant for all species but performed individual runs over a large range of transport times for NH_4_^+^, Na^+^, and Ca^2+^ (Supplementary Fig. 5). Due to the long atmospheric residence time (low wet deposition efficiency) of NH_4_^+^, variation of the transport time has little effect on our glacial/interglacial source reconstruction, strongly corroborating that biogenic emissions were reduced in the North American NH_4_^+^ aerosol source area in addition to a southward shift of vegetation and soil cover due to ice sheet expansion. The same holds true for Ca^2+^, where the assumed low precipitation rates en route also imply a very long atmospheric residence time. For sea salt aerosol (Na^+^), where the residence time is short, the effect of transport time is much larger. Higher glacial sea salt aerosol source concentrations relative to the early Holocene can only be achieved either by short constant mean transport times of around 3 days (not supported by our back-trajectory study) or by increasing transport time in the glacial relative to today as expected from the longer glacial transport distance from the source region to NEEM. Assuming a constant transport time of 5.6 days as today would imply slightly reduced glacial sea salt aerosol emissions, which is highly unlikely in view of higher wind speeds and increased sea ice formation under cold climate conditions. Our best-guess estimate implies about a factor of 1.5 higher sea salt aerosol emissions with a potential range of 1–2. Increases by a factor of 3 are less likely and also reduced glacial source concentrations are still possible within our error limits.

For Ca^2+^ the location of the main source region (predominantely East Asian deserts) is assumed to remain the same for the glacial as for the interglacial as suggested by previous provenance studies^28,29^. Combined with stronger and more zonal jets (which are the main transport pathway for particulate dust and Ca^2+^) during glacial climate conditions, this could lead to decreased glacial transport time and, thus, to more efficient transport. Accordingly, in addition to our best-guess Ca^2+^ run (where transport time was constant at 11.5 days), we performed runs with lower or higher but constant transport time and an alternative run where transport time *t* scaled with temperature, thus, transport during the LGM being only about half as long as during the Holocene. The results of these alternative runs are contrasted with the constant *t* runs in Supplementary Fig. 5. As can be seen in Supplementary Fig. 5, the effect of a reduced glacial transport time leads only to slightly reduced relative source concentration changes. Similar changes are experienced when assuming an aerosol column height of 8 km, i.e., twice as much as in our best-guess run. In contrast, increasing the precipitation rate for Ca^2+^ to 1/3 of the below-cloud value (instead of 1/10 in the best-guess run) reduces the increase in glacial source concentrations substantially to only a factor of 2 compared to the early Holocene. Thus, the assumption on the degree of wet deposition en route, i.e., the atmospheric residence time controlled by wet deposition, is the most critical parameter for our mineral dust aerosol source reconstruction. In view of the modern atmospheric residence time of mineral dust aerosol in our approach being supported by global circulation modelling studies^43^ and because of the agreement of past mineral dust aerosol changes in our reconstruction with data from the northern Pacific^51^, the very low wet dust deposition during transport, as assumed in our best-guess run, appears to reflect atmospheric conditions realistically.

As this is the first time records of ice core impurities cover the entire last interglacial period in Greenland are available, we are now able to compare the current warm period with the Eemian in terms of aerosol concentrations. However, to allow for an unbiased comparison we have to synchronize the Eemian and Holocene sections by shifting the age scales to a common onset of both interglacials. To this end, we superimposed the two warm periods synchronizing the fast CH_4_ increase at the onset of both interglacials^64^. As the NEEM age scale in the Eemian has been derived by synchronizing the NEEM gas records^4^ with respective EPICA Dronning Maud Land (EDML) ice core records and as the NGRIP/NEEM age scales for the Holocene have been previously synchronized to EDML^65^, we can use the EDML CH_4_ record to define a common onset of both the Eemian and Holocene time period (Supplementary Fig. 8). By doing so, also the June insolation at NEEM (showing maxima about 2500 years after the fast methane rise in both periods) and the δ^18^O records (continuously increasing for 2000 years after the methane rise before δ^18^O becomes stable for several thousands of years) are synchronized. Due to the brittle ice, it was not possible to measure the entire Holocene section of the NEEM ice core using our CFA analysis. Accordingly, the windows for the Eemian–Holocene comparison were chosen of the same duration within the periods of stable δ^18^O: 7.6–9.8 kyr BP vs. 124.3−126.5 kyr BP.

### Past accumulation estimates

For the past accumulation rates at NEEM over the last glacial we used the values modelled by ref. ^62^, available for the time period from 108 kyr BP to the present. This accumulation rate was also used to scale the precipitation en route as described above and used in our model reconstruction in Fig. 2 and Supplementary Fig. 4 for this time interval. However, for the Eemian this modelled accumulation estimate is not available. Accordingly, for the comparison of Eemian vs. early Holocene source concentrations in Fig. 3 we had to use a different approach. Note that the source concentrations provided in the supplementary data also use these two different approaches in estimating past precipitation rates for the time interval younger than 108 kyr BP and for the Eemian section. Several studies have determined an empirical relationship between accumulation rate and δ^18^O values of the snow and ice in Greenland. These temperature-δ^18^O-accumulation relationships were determined using various ice cores from different sites on the Greenland ice sheet covering the most recent period^54,66^, or the Holocene and the last glacial period^67,68^. The relationships determined in these studies range from a 8% to 14% increase in accumulation for each permil increase in δ^18^O. Only ref. ^68^ found a stronger dependency of accumulation on δ^18^O during the last glacial of ~17%/‰. However, it remains unclear if this strong relationship remained valid also for earlier warm periods on the Greenland ice sheet. For example an increase in the fraction of summer precipitation due to the strong change in summer insolation during the Eemian^69^ may alter the δ^18^O-accumulation relationship. In the absence of any direct accumulation proxy or model-based results from age scale calculations for the Eemian period, we assume the local spatial δ^18^O-accumulation relationship to be valid also during the Eemian. In line with the location of the NEEM site, we use the relationship as described by Buchardt et al.^54^ for north-west Greenland sites, which represents an intermediate δ^18^O-accumulation sensitivity:

3 $$A = 20.5\, \frac{{{\mathrm{cm}} \, {\mathrm{IE}}}}{{{\mathrm{year}}}}\cdot \mathrm{e}^{0.1\left( {\delta ^{18}\mathrm{O} + 33.7‰}\right)}$$

with *A* being the accumulation rate in cm ice IE per year, 20.5 cm IE/year the modern accumulation rate and −33.7‰ the mean modern δ^18^O value at the NEEM site. In order to account for the entire range covered by the different estimates of the interglacial δ^18^O-accumulation relationships as mentioned above, we use an uncertainty of the Eemian accumulation rate of ±10%, which is considerably larger than the uncertainty indicated in Buchardt et al.^54^. To avoid a systematic bias when comparing Eemian with early Holocene source concentrations in Fig. 3, we used an accumulation rate estimate for the early Holocene which is also based on Eq. (3) for the comparison in Fig. 3. This value is ~10% higher than the modeled accumulation by ref. ^62^. Using Eq. (3) the Eemian accumulation rate is ~25% higher than the early Holocene value.

### Data availability

All data generated or analysed during this study are included in this published arcticle (Supplementary Data 1 and 2). Data are also available on the NOAA paleoclimate and the PANGAEA data base.

## Electronic supplementary material

Supplementary Information

Description of Additional Supplementary Files

Supplementary Data 1

Supplementary Data 2
