# Supplementary material for: Greenland records of aerosol source and atmospheric lifetime changes from the Eemian to the Holocene
Source: Nat Commun. 2018 Apr 16;9:1476. doi: 10.1038/s41467-018-03924-3 (PMC5902614; doi:10.1038/s41467-018-03924-3)
Supplement: Supplementary file 1 — Supplementary Information [file 41467_2018_3924_MOESM1_ESM.pdf]

Supplementary material for

Greenland records of aerosol source and atmospheric lifetime changes from the Eemian to the Holocene

S. Schüpbach[1], H. Fischer[1*], M. Bigler[1], T. Erhardt[1], G. Gfeller[1], D. Leuenberger[1], O. Mini[1], R. Mulvaney[2], N.J. Abram[2,+], L. Fleet[2], M.M. Frey[2], E. Thomas[2], A. Svensson[3], D. Dahl-Jensen[3], E Kettner[3], H. Kjaer[3], I. Seierstad[3], J.P. Steffensen[3], S.O. Rasmussen[3], P. Vallelonga[3], M. Winstrup[3], A. Wegner[4], B. Twarloh[4], K. Wolff[4], K. Schmidt[4], K. Goto-Azuma[5,*], T. Kuramoto[5,**], M. Hirabayashi[5], J. Uetake[5,***], J. Zheng[6], J. Bourgeois[6], D. Fisher[7], D. Zhiheng[8], C. Xiao[8], M. Legrand[9], A. Spolaor[10], J. Gabrieli[10], C. Barbante[10], J.-H. Kang[11], S. D. Hur[11], S.B. Hong[11], H.J. Hwang[11], S. Hong[12], M. Hansson[13], Y. IIzuka[13], I. Oyabu[13], R. Muscheler[14], F. Adolphi[14,1], O. Maselli[15], J. McConnell[15], E.W. Wolff[16]

[*] corresponding author

1 Climate and Environmental Physics, Physics Institute & Oeschger Centre for Climate Change Research, University of Bern, Sidlerstrasse 5, 3012 Bern, Switzerland (email: hubertus.fischer@climate.unibe.ch)

2 British Antarctic Survey, National Environment Research Council, High Cross Madingley Road, Cambridge, CB3 0ET, UK

+ Present address: Research School of Earth Sciences, The Australian National University, Canberra ACT 2602, Australia

3 Centre for Ice andClimate, Niels Bohr Institute, University of Copenhagen, Juliane Maries Vej 30, 2100 Copenhagen K, Denmark

4 Alfred-Wegener-Institut, Helmholtz-Zentrum für Polar-und Meeresforschung, Am Alten Hafen 26, 27568 Bremerhaven, Germany.

5 National Institute of Polar Research, 10-3 Midori-cho, Tachikawa, Tokyo 190-8518, Japan

* SOKENDAI (The Graduate University of Advanced Studies), Shonan Village, Hayama, Kanagawa 240-0193 Japan

** Present address: Fukushima Prefectural Centre for Environmental Creation, 10-2 Fukasaku, Miharu Town, Fukushima 963-7700, Japan

*** Present address: Colorado State University, Department of Atmospheric Science, 200 West Lake Street, 1371 Campus Delivery Fort Collins, CO 80523-1371, USA

6 Natural Resources Canada, Geological Survey of Canada, 601 Booth Street, Ottawa K1A 0E8, Canada.

7 Department of Earth Sciences, Environment and Geomatics, University of Ottawa, Ottawa, ON, Canada
8 State Key Laboratory of Cryospheric Sciences, Cold and Arid Regions Environmental and Engineering Research Institute, Chinese Academy of Sciences, Lanzhou 730000, China
9 Institut des Géosciences de l'Environnement, Université Grenoble Alpes, CS 40 700, 38058 Grenoble Cedex 9, France

10 Institute for the Dynamics of Environmental Processes-CNR, University of Venice, via Torino, 155, 30172 Venice-Mestre, Italy

11 Korea Polar Research Institute, 26 Songdomirae-ro, Yeonsu-gu, Incheon 21990, Republic of Korea
12 Department of Ocean Sciences, Inha University, 100 Inha-ro, Nam-gu, Incheon 22212, Republic of Korea
13 Department of Physical Geography, Stockholm University, S-106 91 Stockholm, Sweden
14 Department of Geology, Lund University, Solvegatan 12, SE-22362 Lund, Sweden.
15 Desert Research Institute, Nevada System of Higher Education, Reno, NV 89512
16 Department of Earth Sciences, University of Cambridge, Downing Street, Cambridge, CB2 3EQ, UK.

	v_{dry} (in m h^{-1})	ε_r (in g air /g rain)	ε_s (in g air /g snow)
Na^{+}	4.1±2.2	600±180	2000±800
NO_3^{-}	2.6±1.9	510±90	2400±500
NH_4^{+}	2.0±1.4	310±140	140±100
SO_4^{-}	0.8±0.7	400±200	220±130
Ca^{2+}	8.3±4.3	1420±1020	840±400

Supplementary Table 1 Model deposition parameters. Typical dry deposition velocities v_{dry} and scavenging ratios in rain en route ε_r and snow ε_s, respectively, and the corresponding uncertainties (from ref.[1-10]).

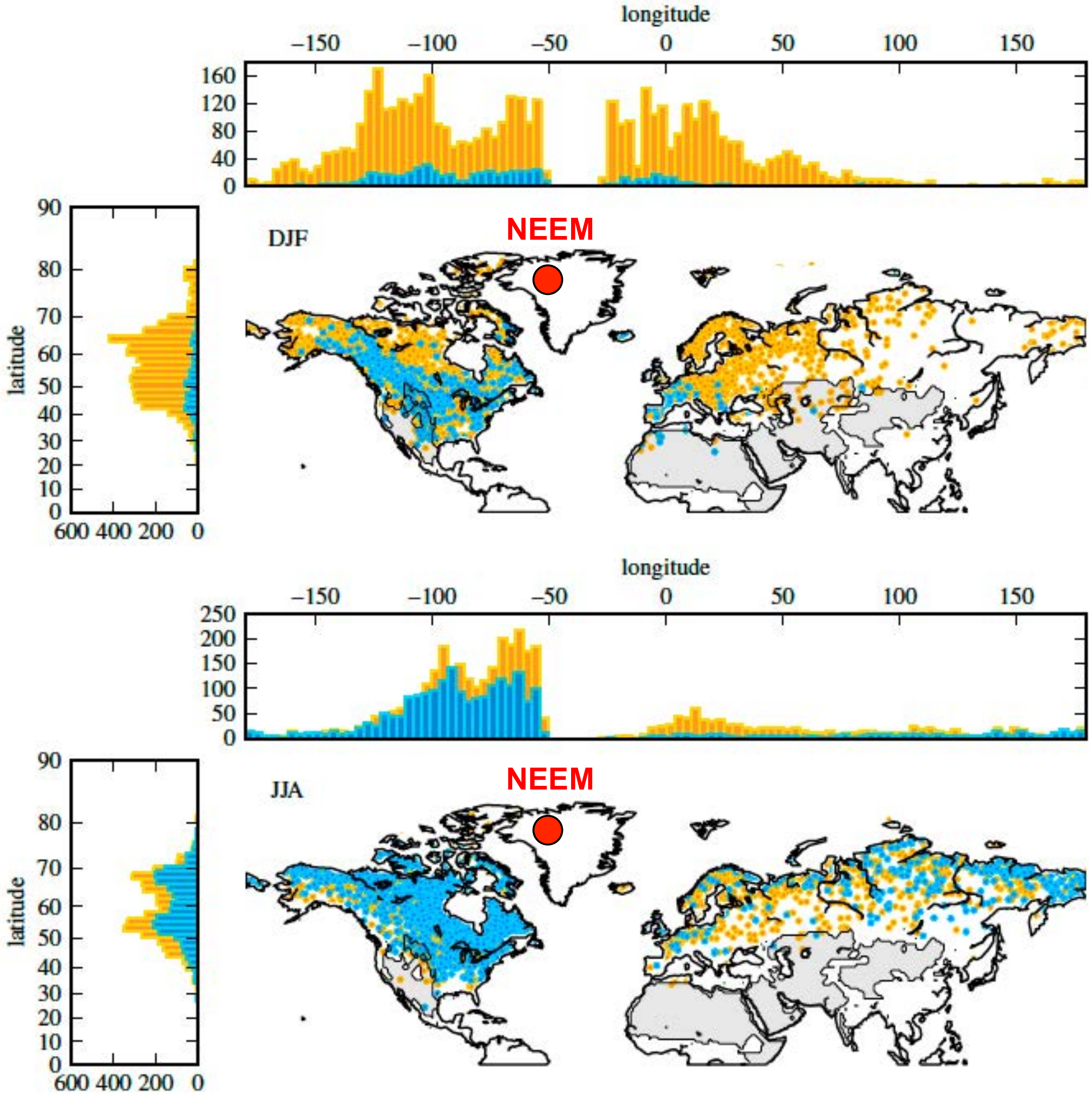

Supplementary Figure 1 Terrestrial back-trajectory analysis. Endpoints of NEEM back-trajectories (using the HySplit model[11] and ERA-interim reanalysis data[12]) and originating from the terrestrial boundary layer for DJF (top) and JJA (bottom). For NH_4^+, summer trajectories are most relevant, when the seasonal NH_4^+ maximum occurs. Boundary layer height at the source is 100 m. Orange dots depict back-trajectories, which started at NEEM under dry conditions, blue dots back-trajectories which started at NEEM when snowfall occurred at NEEM. The grey area depicts arid regions.

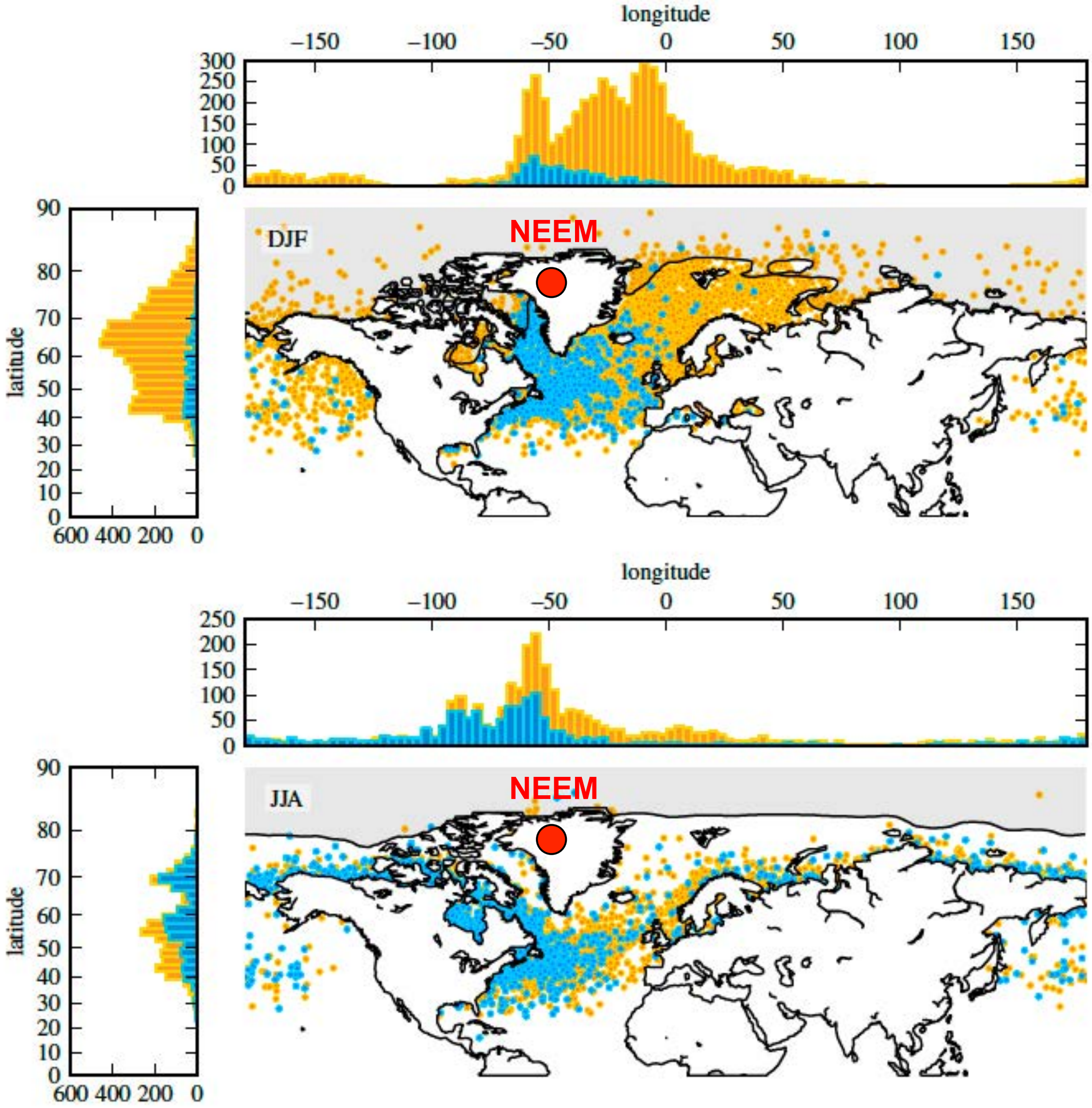

Supplementary Figure 2 Marine back-trajectory analysis. Endpoints of NEEM back-trajectories (using the HySplit model[11] and ERA-interim reanalysis data[12]) originating from the marine boundary layer for DJF (top) and JJA (bottom). For Na^+, winter trajectories are most relevant, when the seasonal Na^+ maximum occurs. Boundary layer height at the source is 100 m. Orange dots depict back-trajectories, which started at NEEM under dry conditions, blue dots back-trajectories which started at NEEM when snowfall occurred at NEEM. The grey area depicts the region where there is more than 90% sea ice.

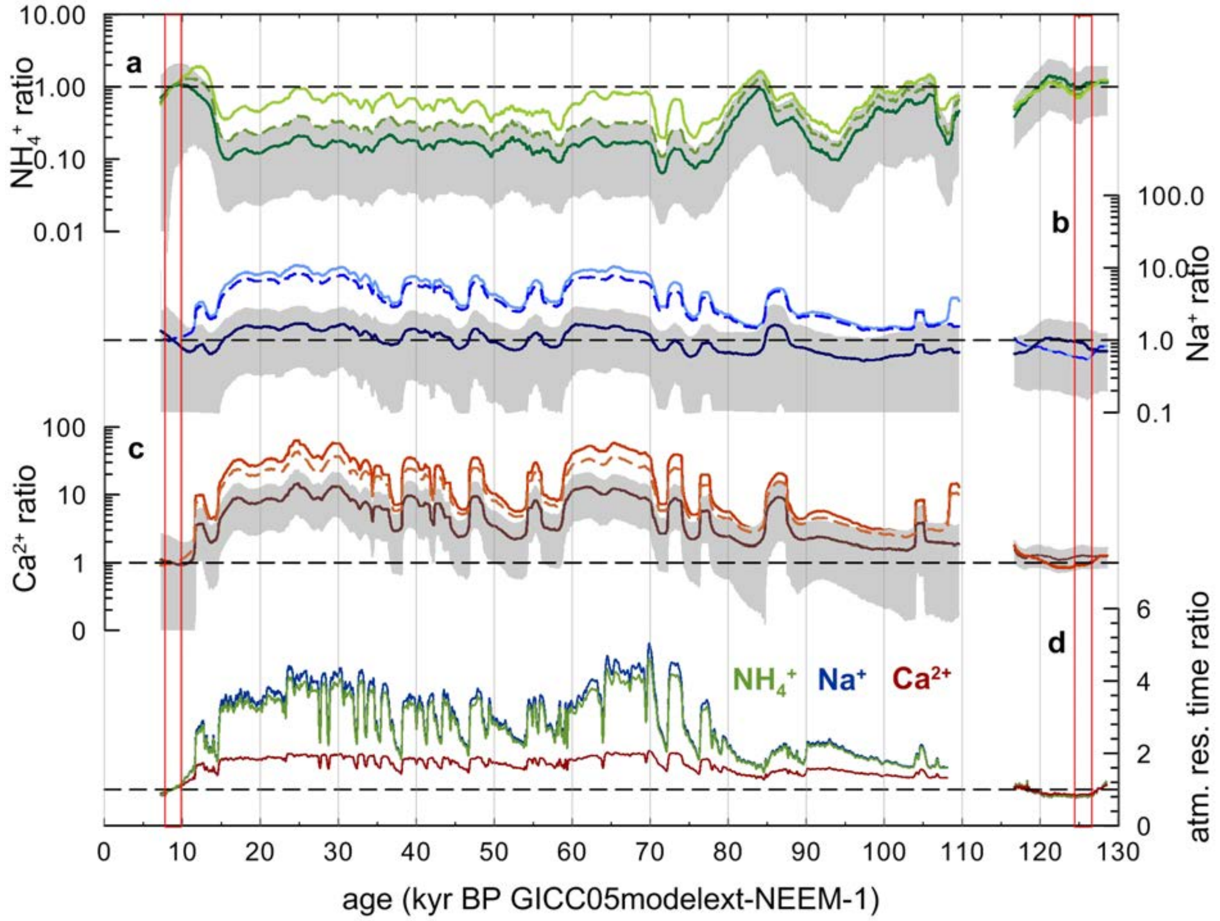

Supplementary Figure 3 Relative concentration changes. Millennial changes (1000 yr running means) of concentrations relative to the early Holocene reference period for a) NH_4^+, b) Na^+, and c) Ca^{2+}. Light solid lines show the relative change in concentration in the ice, dashed lines relative changes in the atmospheric concentration over the ice sheet (correcting for local deposition effects), and dark solid lines the relative changes in the atmospheric concentration at the source (correcting for deposition effects en route) for our best-guess estimate with its uncertainty band (1 sigma) in grey. d) changes in calculated atmospheric residence times for all species relative to the early Holocene value. The red boxes in the early Holocene and early Eemian indicate the periods used for comparison of the relative source changes in Fig. 3.

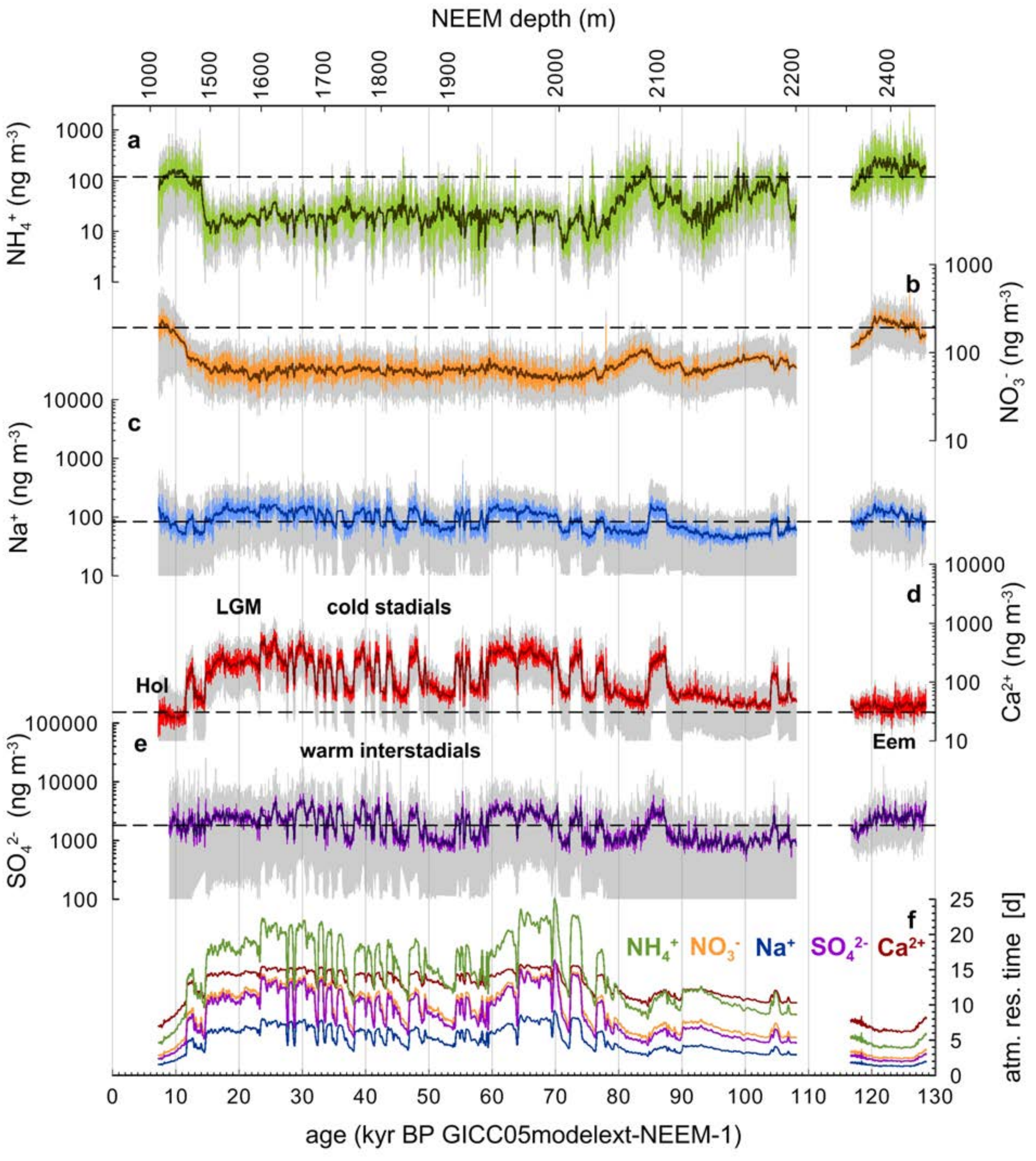

Supplementary Figure 4 Extended suite of reconstructed source concentrations. Variations of the source concentration records in 10 yr resolution (light coloured lines) for a) NH_4^+, b) NO_3^-, c) Na^+ and d) Ca^{2+}. In the case of SO_4^{2-} (e) the light thin line represents a 5 point running mean of the 10 yr data, representative of the maximum 40 yr resolution that can be achieved with the SO_4^{2-} data over the entire length of the record. The dark bold lines represent 21 point running means of the 10 yr data. The grey area represents the uncertainty band (1 sigma) as calculated from Gaussian error propagation of the deposition parameters. Note that the results for NO_3^- and SO_4^{2-} may be biased, as our transport model approach does not account for formation of NO_3^- and SO_4^{2-} from gaseous precursors during transport. For the Eemian section only the uncertainty in the past precipitation rate is included in the uncertainty band, assuming that the other deposition parameters were the same for the Eemian and the Holocene. In panel (f) the calculated atmospheric residence time for each of the aerosol species is plotted.

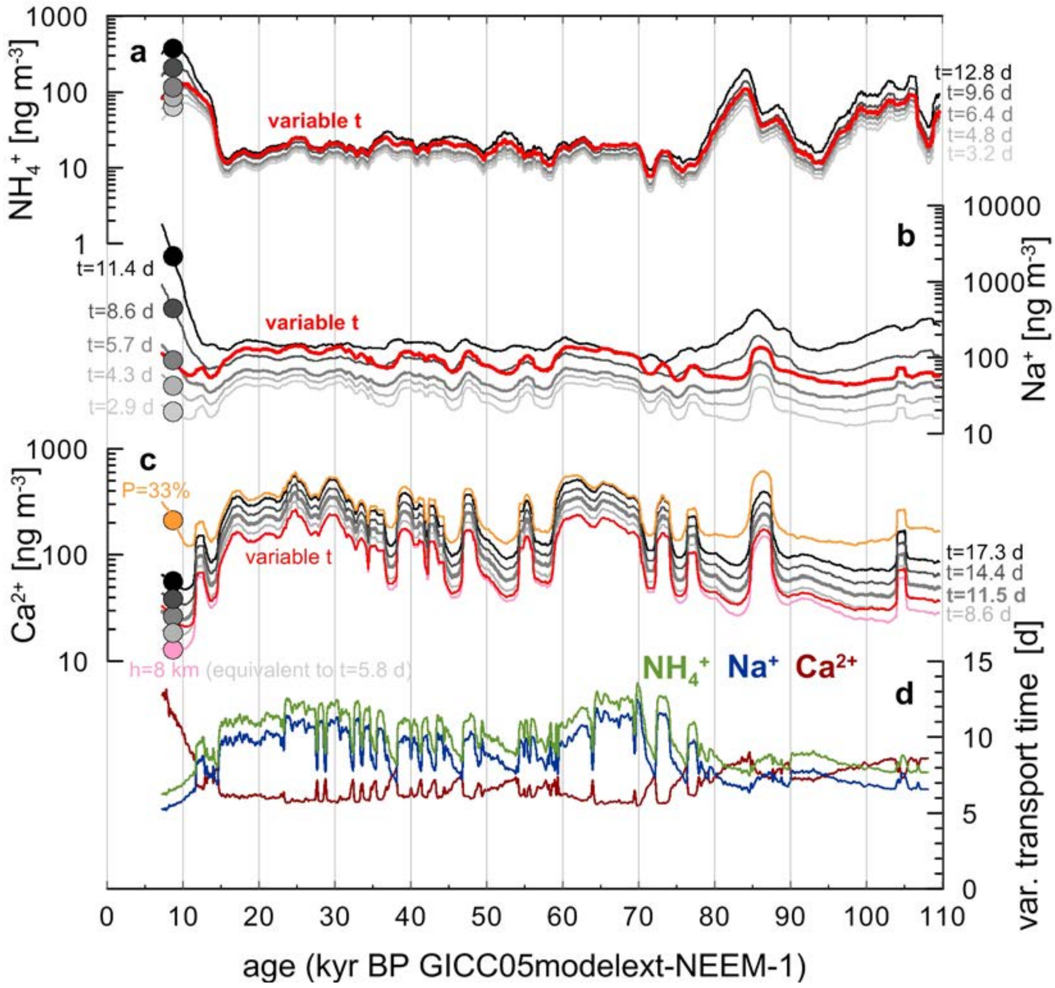

Supplementary Figure 5 Sensitivity runs for source reconstructions. a) influence of transport time on the reconstructed NH_4^+ source concentration. The thin grey lines represent reconstructions for largely differing transport times held constant over time; the red thick line is our best-guess estimate, where transport time is variable and increased by about a factor 2 for glacial times relative to the early Holocene. Note that the sensitivity of the result on the choice of transport time is small for NH_4^+ due to its relatively long atmospheric residence time/inefficient wet deposition. b) same for Na^+. Due to the high scavenging efficiency for sea salt aerosol, the result is very sensitive on the chosen transport time and its temporal evolution. The true atmospheric concentrations at the source are likely to lie in the band defined by t≈5 days and t≈12 days. c) sensitivity runs for Ca^{2+}, where in our best-guess run (grey thick line) transport time is held constant at 11.5 days, h=4000 m and precipitation above cloud level is reduced to 10±10% of the precipitation below cloud level. The thin grey lines represent reconstructions for different transport times held constant over time. The thin red line indicates a run where transport time during the glacial is only about 50% of the Holocene value, the pink line uses the buest-guess parameters except for h=8000 m, the orange line except for precipitation above cloud level being reduced to one third of precipitation below cloud level. Circles on the left indicate the median of the early Holocene values for the respective runs. In d) transport times for the runs with variable *t* are plotted for NH_4^+, Na^+ and Ca^{2+}.

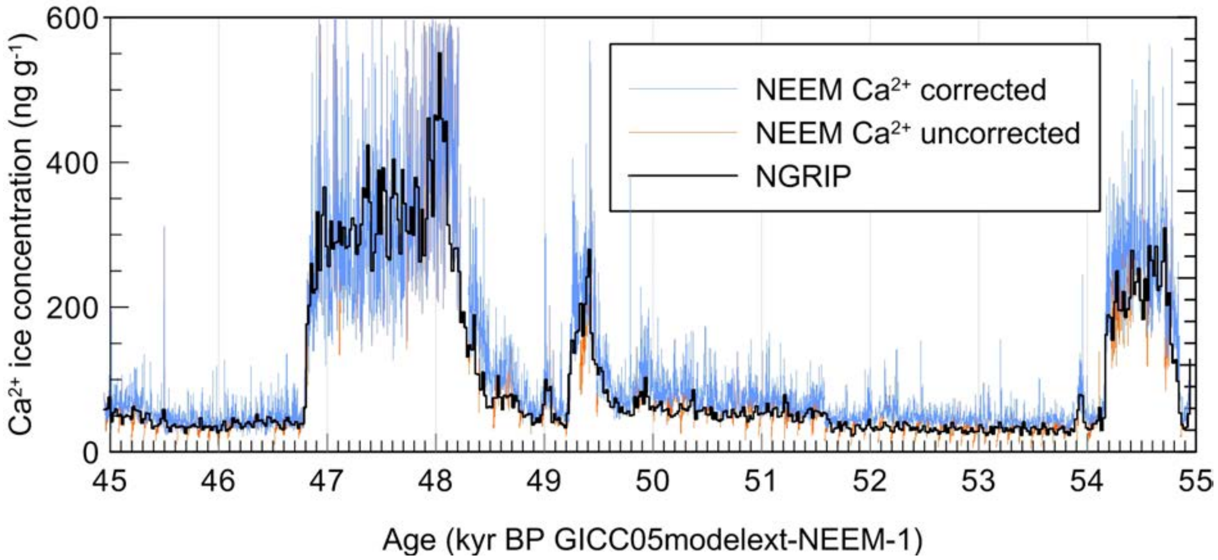

Supplementary Figure 6 Example for the NEEM drill fluid effect on Ca^{2+} concentrations. Typical interval of the NEEM Ca^{2+} record in mm resolution covering stadial and interstadial periods before (orange line) and after (blue line) data correction for the adsorption/desorption effect caused by drill fluid in the CFA system. Each run starts with a negative excursion and then slowly recovers to the true concentration in the ice. Also plotted is the Ca^{2+} concentration of the NGRIP ice core in 20 yr resolution[13] also measured with the Bern CFA system but using another drill fluid, which does not interfere with the CFA analysis. The very good correspondence of the corrected NEEM data and the NGRIP data (taking the different location on the ice sheet and the different accumulation rate into account), shows that decadal Ca^{2+} variations in the corrected NEEM record are closely reflecting true concentrations in the ice.

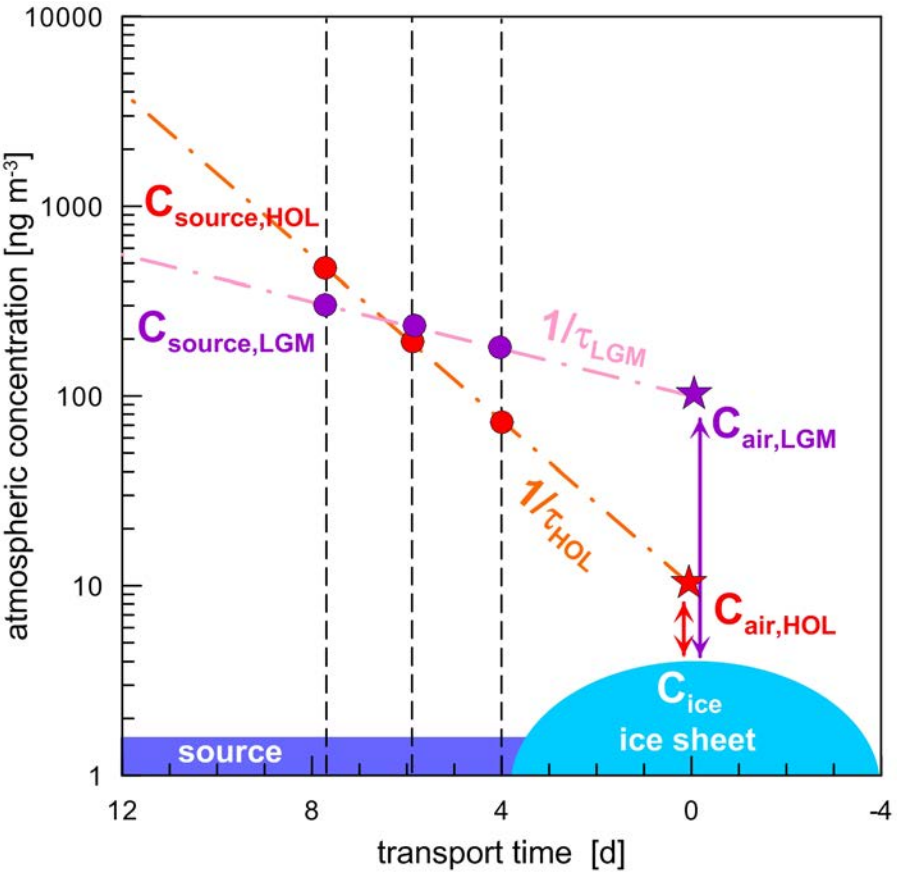

Supplementary Figure 7 Transport model approach. Illustration of the back-calculation of source concentrations from concentrations in the ice and the effect of this deposition correction on glacial concentrations relative to the early Holocene. Atmospheric concentrations over the ice are calculated from concentrations in the ice using equation (1). According to equation (2) the different degree of wet deposition en route for warm and cold conditions leads to different slopes of logarithmic concentrations in the atmosphere along the transport path and, thus, different concentrations at the source. Therefore, the glacial/interglacial ratio of source concentrations changes backwards with transport time t and is strongly dependent on the atmospheric residence time τ.

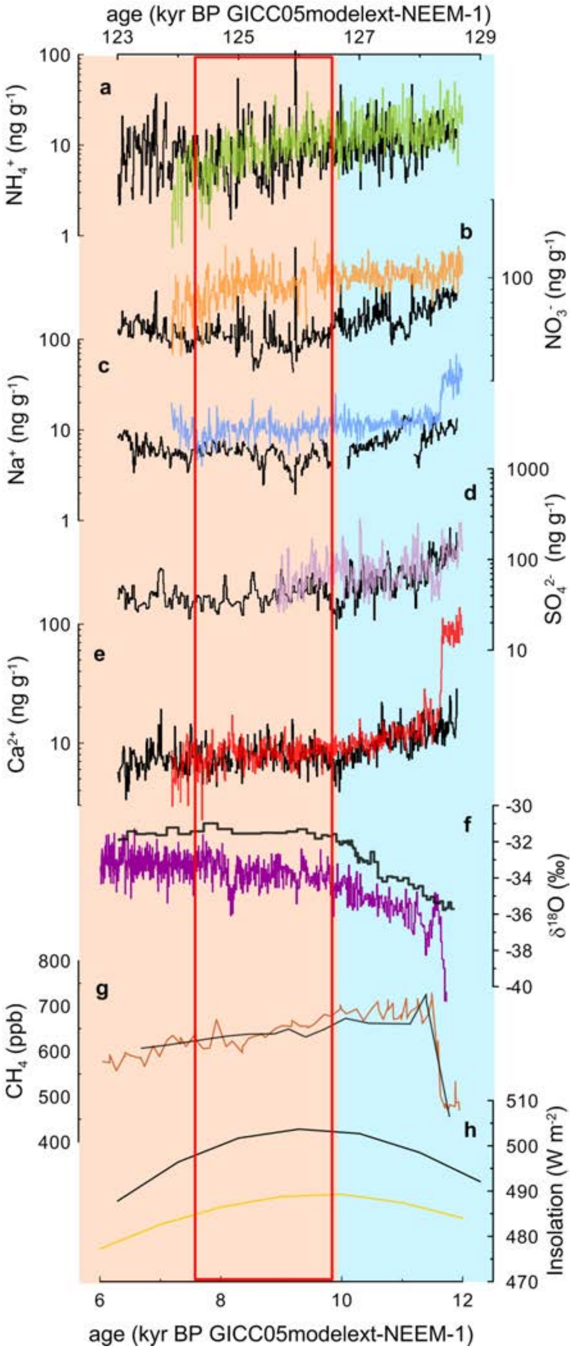

Supplementary Figure 8 Comparison of late termination (blue background) and interglacial (orange background) key climate parameters to define the Eemian and Holocene reference periods. a-e) NEEM aerosol chemistry records, f) NEEM stable water isotope records, g) EDML methane record after synchronisation, h) June insolation at the NEEM site. The early Holocene period (lower x-axis, coloured lines) is overlain by the Eemian period (upper x-axis, black lines) to illustrate the direct comparison of the two interglacial periods. The alignment is done by an optimal fit of the fast methane increase at the transitions into the interglacials and the peak in solar insolation. The most suitable period to compare the two interglacials is indicated by the red box. Unfortunately, the mid-Holocene section is missing due to the brittle ice zone where no CFA could be performed. Therefore, we decided to use the early Holocene (7.6-9.8 kyr BP) and the early Eemian (124.3-126.5 kyr BP) for our direct comparison of the two interglacials.

References

1 Davidson, C. I., Bergin, M. H. & Kuhns, H. D. The deposition of particles and gases to ice sheets, in *Chemical exchange between the atmosphere and polar snow* Vol. 43 *NATO ASI Series* (eds E.W. Wolff & Bales R.C.) 275-306 (Springer Verlag, 1996).

2 Fischer, H., Schüpbach, S., Gfeller, G., Bigler, M., Röthlisberger, R., Erhardt, T., Stocker, T. F., Mulvaney, R. & Wolff, E. Millennial changes in North American wildfire and soil activity over the last glacial cycle. *Nature Geosciences* **8**, 723-727, doi:10.1038/NGEO2495 (2015).

3 Savoie, D. L., Prospero, J. M. & Nees, R. T. Washout ratios of nitrate, non-sea-salt sulfate and sea-salt on Virginia key, Florida and on American Samoa. *Atm Env* **21**, 103-112 (1987).

4 Galloway, J. N., Savoie, D. L., Keene, W. C. & Prospero, J. The temporal and spatial variability of scavenging ratios for NSS sulfate, nitrate, methanesulfonate and sodium in the atmosphere over the North Atlantic. *Atm Env* **27**, 235-250 (1993).

5 Harrison, M. E. & Pio, C. A. A comparative study of the ionic composition of rainwater and atmospheric aerosol. *Atm Env* **17**, 2539-2543 (1983).

6 Kasper-Giebl, A., Kalina, M. F. & Puxbaum, H. Scavenging ratios for sulfate, ammonium and nitrate determined at Mt. Sonnblick (3106 m a.s.l.). *Atm Env* **33**, 895-906 (1999).

7 Barrie, L. A. Scavenging ratios, wet deposition, and in-cloud oxidation: An application to the oxides of sulphur and nitrogen. *J Geophys Res* **90**, 5789-5799, doi:10.1029/JD090iD03p05789 (1985).

8 Buat-Menard, P. & Duce, R. A. Precipitation scavenging of aerosol particles over remote marine regions. *Nature* **321**, 508-510 (1986).

9 Hicks, B. B. A climatology of wet deposition scavenging ratios for the United States. *Atm Env* **39**, 1585-1596 (2005).

10 Bergin, M. H., Jaffrezo, J.-L., Davidson, C. I., Dibb, J. E., Pandis, S. N., Hillamo, R., Maenhaut, W., Kuhns, H. D. & Makela, T. The contributions of snow, fog, and dry deposition to the summer flux of anions and cations at Summit, Greenland. *J Geophys Res* **100**, 16275-16288 (1995).

11 Stein, A. F., Draxler, R. R., Rolph, G. D., Stunder, B. J. B., Cohen, M. D. & Ngan, F. NOAA's HYSPLIT Atmospheric Transport and Dispersion Modeling System. *Bulletin of the American Meteorological Society* **96**, 2059-2077, doi:10.1175/BAMS-D-14-00110.1 (2015).

12 Dee, D. P., Uppala, S. M., Simmons, A. J., Berrisford, P., Poli, P., Kobayashi, S., Andrae, U., Balmaseda, M. A., Balsamo, G., Bauer, P., Bechtold, P., Beljaars, A. C. M., van de Berg, L., Bidlot, J., Bormann, N., Delsol, C., Dragani, R., Fuentes, M., Geer, A. J., Haimberger, L., Healy, S. B., Hersbach, H., Hólm, E. V., Isaksen, L., Kållberg, P., Köhler, M., Matricardi, M., McNally, A. P., Monge-Sanz, B. M., Morcrette, J. J., Park, B. K., Peubey, C., de Rosnay, P., Tavolato, C., Thépaut, J. N. & Vitart, F. The ERA-Interim reanalysis: configuration and performance of the data assimilation system. *Quarterly Journal of the Royal Meteorological Society* **137**, 553-597, doi:10.1002/qj.828 (2011).

13 Rasmussen, S. O., Bigler, M., Blockley, S. P., Blunier, T., Buchardt, S. L., Clausen, H. B., Cvijanovic, I., Dahl-Jensen, D., Johnsen, S. J., Fischer, H., Gkinis, V.,

Guillevic, M., Hoek, W. Z., Lowe, J. J., Pedro, J. B., Popp, T., Seierstad, I. K., Steffensen, J. P., Svensson, A. M., Vallelonga, P., Vinther, B. M., Walker, M. J. C., Wheatley, J. J. & Winstrup, M. A stratigraphic framework for abrupt climatic changes during the Last Glacial period based on three synchronized Greenland ice-core records: refining and extending the INTIMATE event stratigraphy. *Quat Sci Rev* **106**, 14-28, doi:10.1016/j.quascirev.2014.09.007 (2014).
