# Supplementary material for: Greenland records of aerosol source and atmospheric lifetime changes from the Eemian to the Holocene
Source: Nat Commun. 2018 Apr 16;9:1476. doi: 10.1038/s41467-018-03924-3 (PMC5902614; doi:10.1038/s41467-018-03924-3)
Supplement: Supplementary file 2 — Description of Additional Supplementary Files [file 41467_2018_3924_MOESM2_ESM.pdf]

Description of Additional Supplementary Files

File Name: Supplementary Data 1
Description: NEEM ion concentration data in 10 yr resolution.

File Name: Supplementary Data 2
Description: Aerosol source concentrations and atmospheric lifetime in 10 yr resolution derived from the NEEM ice core data.
